# A concept for the molecular design of readily treatable chemicals

**DOI:** 10.1039/d5su00944h

**Published:** 2026-03-20

**Authors:** Gabriel Sigmund, Sanne J. Smith, Anett Georgi, Thomas V. Wagner, Mohamed Ateia, Benedikt M. Aumeier, Jouke E. Dykstra, Holger V. Lutze, Michael Neumann, Nora B. Sutton, Daniel Zahn, Zhanyun Wang

**Affiliations:** a Environmental Technology, Wageningen University P. O. Box 17 6700 AA Wageningen The Netherlands gabriel.sigmund@wur.nl; b Department of Water Management, Delft University of Technology Stevinweg 1 2628 CN Delft The Netherlands; c Department of Technical Biogeochemistry, Helmholtz Centre for Environmental Research – UFZ Leipzig Germany; d Department of Chemical and Biomolecular Engineering, Rice University Houston TX USA; e Chair of Urban Water Systems Engineering, Technical University of Munich Am Coulombwall 3 85748 Garching Germany; f Technical University of Darmstadt, Institute IWAR, Chair of Environmental Analytics and Pollutants Franziska-Braun-Straße 7 64287 Darmstadt Germany; g IWW Water Centre Moritzstraße 26 D-45476 Mülheim an der Ruhr Germany; h Centre for Water and Environmental Research (ZWU) Universitätsstraße 5 D-45141 Essen Germany; i German Environment Agency, Section IV 2.3 Chemicals Wörlitzer Platz 1 06844 Dessau-Roßlau Germany; j Department of Environmental Analytical Chemistry, Helmholtz Centre for Environmental Research – UFZ Leipzig Germany; k Empa-Swiss Federal Laboratories for Materials Science and Technology, Technology and Society Laboratory 9014 St. Gallen Switzerland; l National Centre of Competence in Research (NCCR) Catalysis ETH Zürich 8093 Zürich Switzerland

## Abstract

Existing chemicals assessment and management approaches focus on chemical behavior in the natural environment and humans, including using chemical-specific inherent properties such as persistence (P), bioaccumulation potential (B), and mobility (M). To prevent chemical pollution, concepts such as safe-by-design and benign-by-design consider P, B, M, toxicity, and other hazardous properties when selecting existing chemicals or developing new ones. However, certain applications rely on chemical properties that inherently conflict with safe-by-design (*e.g.*, high stability during use often results in P). In such cases, in addition to applying such chemicals only for essential uses and reducing emissions, early consideration of effective removal using available (water) treatment technologies may also be advisable. This may serve as a second line of defense to safe-by-design by minimizing environmental exposure. Here we explore inherent chemical properties relevant to “treatability”, focusing on commonly used and widely available water treatment technologies. These technologies include (i) biodegradation in wastewater and drinking water treatment, (ii) advanced separation technologies such as activated carbon and membrane-based separation, and (iii) oxidation processes. Our conceptual framework sheds light on “treatable-by-design” chemicals for specific applications where safe-by-design chemicals are not (readily) feasible, offering potential for further exploration by the broader community.

## Abstract 2

A new framework to identify “readily treatable” chemicals based on molecular features relevant to water-treatment technologies, can complement “safe-by-design” approaches. Image generated with BioRender.

## Graphical abstract

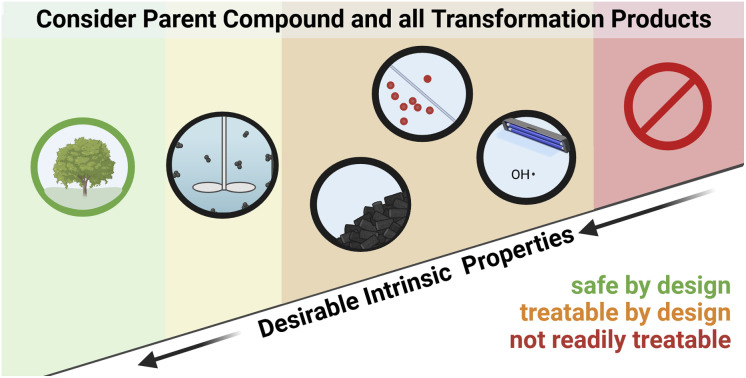

**Sustainability spotlight**

This work supports sustainability by providing a framework to identify and design “readily treatable” chemicals based on molecular features relevant to water treatment technologies, complementing “safe-by-design” approaches. This is important because certain essential applications rely on chemicals properties that inherently conflict with safe-by-design (*e.g.*, high stability resulting in persistence). In these cases, considering their effective removal using available (water) treatment technologies is advisable and can serve as a second line of defence for human and ecosystem health by minimizing exposure and spread of such chemicals. Our conceptual framework enables the identification of safer chemical alternatives, supporting responsible design, production and use (SDG 12), facilitating chemicals removal during water treatment (SDG 6) and protecting human and ecosystem health (SDG 3).

## Introduction

As the global demand for safe and clean water continues to rise, we face challenges associated with the depletion of freshwater resources due to overexploitation, insufficient wastewater treatment, the climate crisis, and chemical pollution. Over the past century, the number and volume of anthropogenic chemicals have continuously increased, encompassing over 350 000 different chemicals and mixtures of chemicals registered globally.^1^ A substantial fraction of these chemicals are emitted directly or indirectly to water bodies and can cause detrimental effects on human and ecosystem health. Safeguarding and improving water quality is of seminal importance for both societies and ecosystems worldwide, beginning with pollution prevention.

Current chemicals assessment, management, and regulation are largely centered around their environmental fate and their potential to interact with biota, including toxicity (T) and bioaccumulation (B). For example, persistence (P) and mobility (M) are chemical-specific inherent properties, quantified by the degradation half-live (DT_50_) of a chemical in soil, sediment, and water, and its organic carbon-normalized sorption coefficient (*K*_oc_), respectively. The combination of these two parameters has proven to be valuable in describing and predicting the environmental fate of chemicals and are widely used in chemicals assessment and regulation around the world.^2–4^

Notably, the assessment and management of chemicals is shifting from a reactive to a proactive approach. Concepts such as “safe-by-design”, “benign-by-design” and “safe-and-sustainable-by-design” have been established and promoted, integrating considerations of P, M, B, T, and other hazardous properties to select existing chemicals and develop new ones that are non-hazardous by design for production and use, aiming to minimize their potential to cause adverse effects on human and ecosystem health.^5–8^ Additionally, the transition to safe-and-sustainable-by-design alternatives may go beyond chemical substitution by involving changes in application and use, as illustrated by the example of modifying metal plating processes to eliminate the need for perfluorooctanesulfonic acid (PFOS) in the first place.^9^ These innovative, forward-looking concepts and frameworks empower chemical and process designers with the tools needed to select nonhazardous, fit-for-purpose chemicals, thereby preventing future harms.

One important challenge that these current concepts and frameworks may not fully resolve is the reliance of certain essential applications on chemical-specific inherent properties, which may be intrinsically hazardous. For example, in some industrial or medical applications, a chemical must possess a high chemical stability to fulfill specific functional requirements, which can translate into a high persistence. To make informed choices and reduce risks, in these specific cases, it becomes critical to understand how effectively these chemicals can be treated using available (water) treatment technologies. This should happen at the chemical design and selection stage to avoid causing unintentional contamination of the natural environment and water sources.

To complement existing approaches, we propose the concept of “treatable-by-design”. Importantly, integrating treatability considerations in molecular design should be considered a second line of defense for protecting human and ecosystem health. This concept is particularly relevant in cases where concepts such as “safe-by-design”, “benign-by-design” and “safe-and-sustainable-by-design” cannot be fully applied due to technical requirements and constraints.

**Fig. 1:**
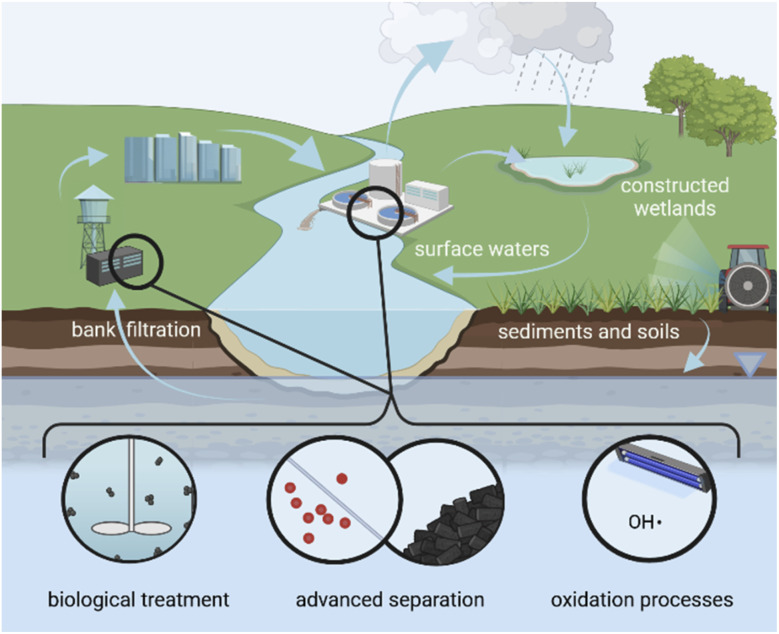
Representation of chemical pollution in a water cycle with multiple barriers. (i) Nature-based barriers and technologies (see text in white) can be well-reflected by chemical-specific inherent properties persistence (P) quantified by DT_50_ and mobility (M) quantified by *K*_oc_, whereas (ii) advanced technologies can only be inadequately reflected by these two environmental fate parameters (black circles).

To enable treatable-by-design, it is critical to understand how treatability of a chemical can be accurately parameterized. Ideally, treatability concepts and parameters would be linked to the inherent properties of a chemical, allowing for easy comparison across different matrices, as is the case for P and M. Indeed, treatability by some nature-based treatment technologies, such as riverbank filtration and soil passage, can be described using P and M if the experimental parameters DT_50_ and *K*_oc_ are available.^10^ This is not the case for many engineered water treatment technologies (see black circles in Fig. 1). This issue could be overcome by complementing P and M with additional treatability indicators.

While designing chemicals for biological treatment is well aligned with the “design for degradation” principle (principle #10 of the twelve principles for green chemistry),^11^ such clear principles do not yet exist for separation techniques and oxidation processes. Building on existing frameworks and knowledge, a well-developed framework of treatable-by-design may facilitate evidence-based decision-making, such as best selecting existing chemicals based on their susceptibility to removal during water treatment. Additionally, although some previous studies have investigated ways to predict the treatability of chemicals,^8,12^ they typically focus on specific technologies, or specific aspects of given technologies. Also, data driven models, such as machine learning, are often developed and used without the mechanistic understanding required for designing readily treatable chemicals.

To enable a comprehensive integration of treatability considerations into chemical design, assessment, and regulation, we here present a conceptual framework that goes beyond previously proposed data-driven frameworks on chemicals across their life cycle,^8^ building upon a mechanistic exploration of the inherent properties of chemicals that determine their treatability in a set of commonly applied and widely available water treatment technologies. These technologies include (i) biodegradation in wastewater and drinking water treatment, (ii) advanced separation technologies, such as activated carbon and membrane-based separation, and (iii) chemical oxidation processes. We explore the state of knowledge in separate sections for each of these technologies and end each section by identifying key takeaways for chemical selection and design, as well as proposing potential experimental cutoff values to operationalize the “treatable-by-design” concept. We further propose a hierarchy of technologies in the consideration of treatable-by-design.

## Treatability *via* biodegradation during water treatment

Biodegradation – the enzymatic transformation of chemicals by microorganisms – is essential for their removal in many water treatment systems, including conventional activated sludge, anaerobic digestion, biological (granular) activated carbon, and rapid and slow sand filters. In water resources and treatment systems, many concerning anthropogenic organic chemicals typically occur in the ng L^−1^ to µg L^−1^ range, and are referred to as organic micropollutants (OMPs) hereafter. Water treatment systems are neither designed, nor commonly operated, with the aim of removing OMPs. Rather, biodegradation occurs concomitantly with biotransformation of other more abundant constituents, such as organic matter and nitrogen species, *i.e.* co-metabolic degradation. Co-metabolic OMP degradation occurs because OMP concentrations are too low to sustain dedicated degrading microorganisms. Examples of such conversions are heterotrophic conversions, such as denitrification, methanogenesis, and DOC degradation, as well as autotrophic conversions, such as nitrification and methane, iron-, and manganese oxidation. The water treatment systems in which these processes occur are engineered to select for microbial communities with specific biological functions for the defined purposes, by adjusting redox conditions, hydraulic retention times, sludge retention times, biofilm carriers, backwashing regimes, substrate loading rates, and empty bed contact times.^13^

Treatability of an OMP in water treatment systems *via* biodegradation depends greatly on the specific conditions within a given treatment. It is important to stress that the occurrence of OMP biodegradation in these treatment technologies does not necessarily imply that OMPs are completely mineralized. Instead, OMP biodegradation frequently results in the transformation of OMPs into transformation products with similar molecular structures as the initial parent chemical. These OMP transformation products can be more hazardous than the initial parent chemical.^14^ In the following paragraphs, “degradation” and “mineralization” are used to distinguish transformation from complete mineralization.

### Current methods and tools for chemical design and their gaps

The biodegradability of a chemical is typically expressed as degradation half-live (*i.e.* the degradation time (DT) required for 50% degradation of the chemical in specific environmental media, DT_50_). Experimentally, DT_50_ values can be derived from standardized tests to assess the environmental persistence of chemicals, such as those from the OECD, EPA, ASTM or ISO.^15^ The most widely used testing guidelines are those from the OECD, which prescribe testing the biodegradability by adding a given chemical to a simulated environmental medium such as activated sludge, sediment, or water. The specific experimental set-up is adjusted to the compartment of interest. For instance, the biodegradability in activated sludge (OECD 303A) is tested in a reactor set-up with microorganisms obtained from a wastewater treatment plant.^16^ The chemical removal is subsequently monitored over the course of weeks to months, after which DT_50_ values are derived. However, these DT_50_ rarely predict half-lives in water treatment systems adequately. This is because these simplified tests cannot sufficiently represent water treatment systems. For example, OECD 303 tests biodegradation in activated sludge under fully aerobic conditions. However, activated sludge systems often include a sequence of redox conditions, to support denitrification and biological phosphorous removal.^13^ Furthermore, OECD tests focus on planktonic degradation in watery slurries, while water treatment systems also include biomass present on the solid matrix in biofilms, which can have different biodegradation dynamics. The adjustment of existing biodegradability tests, or developing completely new test methods, is very time consuming and unlikely to capture the wide variety in possible treatment conditions (including microbial composition, redox conditions, residence time, and co-substrate composition).

Meanwhile, the understanding of molecular features-biodegradability relationships form the basis of Quality Structure Activity Relationships (QSAR) to predict the degradability of a chemical and the formation of persistent transformation products. QSAR tools to predict degradation and transformation product formation include the freely available BioWin,^17^ EnviPath,^18^ and Biotransformer^19^ tools, which can help screen for persistent chemicals, including transformation products. Recently, the EnviPath team has released the “EAWAG Sludge package” for predicting a chemicals degradation in sludge, which is a big step forward in screening for persistence in biological water treatment. Still, models that take into account concentration, co-substrate, and process related parameters in water treatment are largely missing.^20^ Similarly, approaches to assessing and predicting full mineralization and/or the formation of persistent transformation products are missing, but necessary to ensure no persistent transformation products are formed during a given process.^14^ In addition, an inherent uncertainty relating to the complexity of biodegradation and the variability in experimentally derived DT_50_ values used to train such models cannot be overcome with current computational approaches.

### Insights for chemical design

Batch experiment-derived DT_50_ as well as QSAR based screening tools can inform selection of existing chemicals and aid in the design of new chemicals. Existing tools^18,21^ can inform on the expected degradability as well as the type of likely transformation products for a given chemical. Parent compounds and transformation products should be screened for persistence and potential toxicity. As of now, these tools should, however, be seen as screening aids for prioritization rather than final evidence. Curated browsers for experimentally confirmed transformation products, such as (https://fairtps.lcsb.uni.lu/)^22^ can inform read-across for likely transformation products of given chemicals, complementing more uncertain predictive tools. Further improvements in the prediction of treatability in biological treatment at differing water chemical conditions combined with a better understanding of read-across opportunities of experimental insights across environmental compartments^23^ will improve reliability and confidence to inform future chemical selection and design. However, promising candidates should always be experimentally vetted to ensure mineralization or formation of only safe transformation products at relevant conditions. Therein, a treatment system-specific DT_50_ of a few days or less could be set as an indicative cutoff to ensure sufficient degradation of a chemical during biological treatment, as typical residence times in such systems range from hours to days. This guidance is directly related to the Green Chemistry Principle 10: design for degradation. In contrast, all subsequent criteria are conceptually distinct from this established principle.

**Fig. 2:**
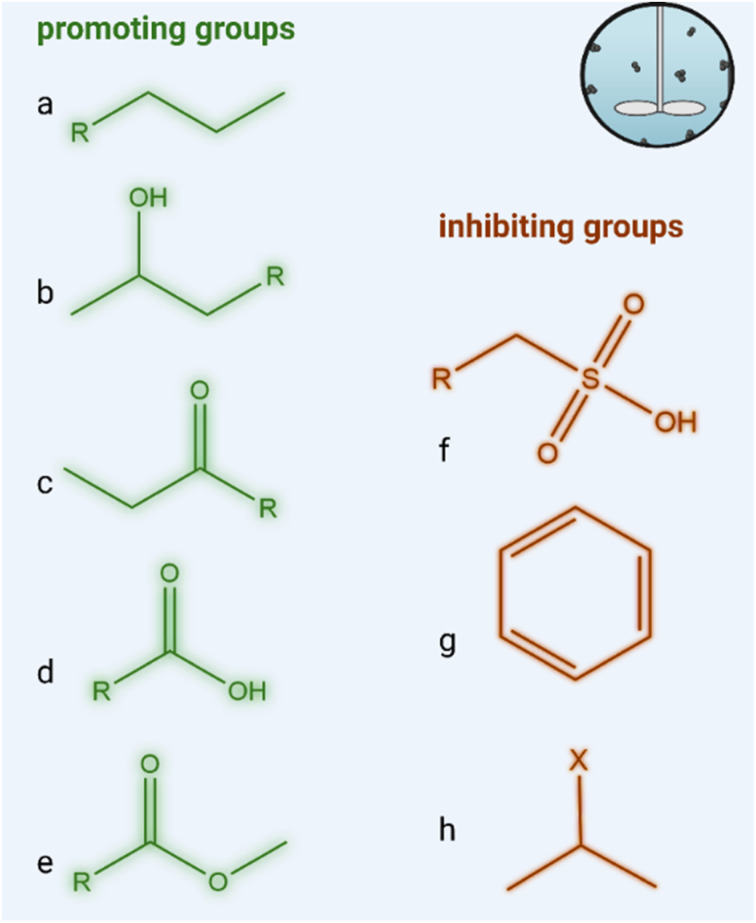
Example of functional groups promoting biodegradability (green) and inhibiting biodegradability (brown) under aerobic conditions. (a) Long unbranched alkyl chains, (b) hydroxyl group, (c) carbonyl group, (d) carboxyl group, (e) ester, (f) sulfonic acid, (g) aromatic ring structure (h) halogenated structure.

Based on current understanding, the following rules of thumb may already be proposed to guide future chemical design for enhanced biodegradability. Molecular features that promote the aerobic biodegradation of a chemical include long unbranched alkyl chains, hydroxyl groups attached to a chain structure, and one or more carbonyl, carboxyl, or ester groups attached to either a chain or ring structure (Fig. 2).^24^ These are all susceptible to oxidation by aerobic microbial enzymatic processes.^25–27^ For ionizable compounds, the neutral fraction is more bioavailable for biodegradation than the ionized fraction.^28^ In contrast, features that may inhibit the biodegradability of a chemical include aromatic ring structures, halogenated structures, and sulfonic acids.^26,27^

## Treatability *via* advanced separation technologies

**Fig. 3:**
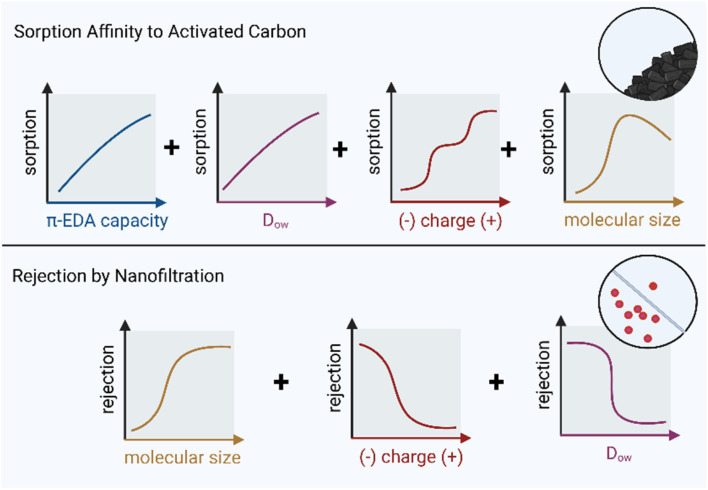
Relationships between molecular features and treatability *via* advanced separation technologies. Top: π-electron donor–acceptor capacity (π-EDA capacity), pH-dependent hydrophobicity (*D*_OW_), polarity and charge, and molecular size of a chemical all affect its sorption affinity towards activated carbon. Bottom: the molecular size, charge, and pH-dependent hydrophobicity (*D*_OW_) of a chemical all affect its rejection by nanofiltration membranes.

Separation approaches can be split into adsorbent-based separation, including activated carbon (AC) and ion exchange resins (IER), as well as membrane-based approaches such as reverse osmosis (RO) and nanofiltration (NF). While some chemical molecular features are linked to high or low removal across these technologies, other features are distinctively relevant only for some of them (Fig. 3).

## Adsorbent-based separation

AC is the most widely used adsorbent for removing OMPs from water. It is a highly carbonaceous solid (C > 80%) characterized by a complex pore system, with a high specific surface area based primarily on micropores (<2 nm) and mesopores (2–50 nm). AC surfaces are rather non-polar and highly aromatic with condensed polyaromatic regions in the basal planes. When in contact with water, AC acquires surface charges through proton adsorption on its polyaromatic regions and through protonation/deprotonation equilibria of heteroatom-containing functional groups, such as those with nitrogen or oxygen. These features provide AC with a high sorption affinity for organic compounds. The driving forces for adsorption on AC are the hydrophobic effect as well as OMP-surface interactions, including π–π electron–donor–acceptor interactions, electrostatic interactions, ion exchange, and (charge-assisted) H-bonding.^29^

Ion exchange resins are also widely used adsorbents and include negatively charged cation exchange resins and positively charged anion exchange resins. Anion exchange resins are capable to bind even small aliphatic and negatively charged OMPs that can hardly be adsorbed by AC. However, ion exchange resins are not very selective, which results in them being quickly saturated by other “non-target” competing ions, including inorganic salts and natural organic matter. Thus, while ion exchange resins can be very useful to remove ions from water, their use in water treatment can only be economically and environmentally operable when paired with another treatment technology that can first remove the majority “non-target” compounds from water (salts, organic matter, other ionic moieties). Additionally, if molecules also bind to the resins by non-electrostatic interactions, regeneration can be challenging.^30^ Therefore, our analysis of treatability *via* adsorbent-based separation is primarily focused on AC.

### Current methods and tools for chemical design and their gaps

AC has a higher sorption affinity for OMPs than organic matter in soils. Sorption to AC at environmentally relevant concentrations is typically at least one order of magnitude stronger than that to organic matter (*K*_oc_), but is not necessarily proportional,^31^ which renders the predictions of treatability *via* AC based on M unreliable. Three main qualitative differences between the two sorbents are: (i) soil organic matter mainly sorbs chemicals through the hydrophobic effect in its more polymeric, “rubbery” absorbent phase and can contain few adsorptive sites, whereas AC acts as a “glassy” adsorbent. (ii) The higher heteroatom content and water swelling of soil organic matter result in a less suitable sorption environment for hydrophobic compounds than the non-polar surfaces in the close-fit micropores of AC. (iii) The higher aromaticity of AC provides substantially more opportunities for π–π electron–donor–acceptor interactions. These more favorable entropic and enthalpic contributions to adsorption on AC can better compensate for potential free energy penalties, such as those caused by unfavorable electrostatic interactions during the adsorption of ionic organic compounds. Therefore, hydrophobicity (*K*_OW_ or *D*_OW_) is of limited value for estimating sorption affinity to AC, especially for ionic and/or ionizable organic compounds, such as many pharmaceuticals, industrial chemicals, and some pesticides, as different processes drive sorption to AC.^32,33^

The scientific community has made efforts to develop predictive tools for sorption to AC based on poly-parameter linear free energy relationships,^34,35^ and neural networks.^36^ These tools utilize molecular solute descriptors such as sorbate molar volume, hydrogen bonding affinities and polarizability, as well as charge-specific descriptors. Some models also include sorbent descriptors, such as surface area and H/C ratio, to predict sorption across different ACs.^36^ However, the predictive power of these tools is still limited due to insufficient experimental sorption data coupled with high-quality sorbent characterization data, both of which are necessary for robust model training. Additionally, there is still a lack of widely accepted and available compound descriptors for ionic organic compounds.

### Insights for chemical design

Low molecular volume, absence of aromatic rings, high heteroatom content and (multiple) negatively charged functional groups, are detrimental for adsorption to AC.^29,33,36,37^ Consequently, high hydrophobicity, presence of aromatic rings, and absence of negatively charged functional groups tend to favor treatability of a given chemical *via* AC. To ensure adequate removal by AC and that breakthrough only occurs only after months of AC column operation, the sorption affinity (*K*_d_) determined from sorption batch experiments should exceed hundreds of L kg^−1^, corresponding to log *K*_d_ > 2. Unfortunately, soil derived *K*_oc_ values are not good predictors for sorption affinity to AC, so batch experiments using AC are advised for assessment.^38^

## Membrane-based separation

Reverse osmosis (RO) and nanofiltration (NF) are membrane-based separation processes. During separation, a pressurized feed stream is introduced into a module containing a membrane, allowing water to permeate through while retaining solutes. As a result, a permeate solution is produced, with a concentrated fraction of solutes remaining in the concentrate stream. RO membranes are highly effective in rejecting most solutes, whereas NF membranes selectively reject specific fractions of solutes. Generally, RO can remove almost all OMPs except for small, uncharged molecules. The resulted concentrate stream may be of concern and requires further treatment or disposal. Since RO retains all solutes, including monovalent ions, addressing the concentrate stream in subsequent treatment steps can pose significant challenges, in addition to the substantially higher energy costs of RO over NF.^39^ Thus, our analysis of treatability *via* membrane-based separation focuses on NF.

### Current methods and tools for chemical design and their gaps

The removal or rejection of chemicals by NF membranes depends on various factors, including membrane characteristics, solvent–solute interactions, and solute properties.^40^ Larger components are sieved by the membrane based on their size relative to the pore size of the membrane. Membrane manufacturers often specify a molecular weight cut-off (MWCO) of their membranes, which represents the lowest molecular weight (MW) at which 90% of solutes are rejected by the membrane. Molecular weight is typically used as a proxy for molecular size; however, the presence of heavier elements in an organic molecule (*e.g.* Zn or Br) can somewhat distort the relationship between size and weight. Further, the pH-dependent speciation and related charge state of ionic or ionizable organic chemicals can be used to determine the electrostatic separation of target chemicals. Additionally, the solubility and hydrophobicity of a chemical can be used to parameterize its aversion against water. Of course, the rejection of chemicals is influenced not only by the membrane properties and a chemicals characteristics, but also by the solution composition and operational conditions of the process.^41^

### Insights for chemical design

Negatively charged chemicals and larger molecules are effectively rejected by typically negatively charged NF membranes.^42,43^ Conversely, the rejection of uncharged species is generally low, particularly for compounds with MW < MWCO.^42,44^ Uncharged components with MW > MWCO and hydrophilic characteristics typically show moderate to high rejection rates.^42,43^ To ensure adequate removal in common NF setups, a MWCO of at least 300 should be considered as an indicative cutoff value.

## Treatability *via* oxidation processes

Oxidation processes such as ozonation, chlorination and chlorine dioxide-based oxidation are used for disinfection and OMP degradation *via* direct oxidation. In contrast, advanced oxidation processes are based on the *in situ* generation of hydroxyl radicals (˙OH, with “˙” indicating an unpaired valence electron) from a primary oxidant that is introduced into the water and sometimes activated using catalysts.^45^ For example, OH-radicals are generated in the Fenton reaction (Fe(ii) and hydrogen peroxide) or photolysis of hydrogen peroxide. These OH-radicals in turn react with most compounds at nearly diffusion-controlled reaction rates (*i.e.*, *k* ∼10^9^–10^10^ M^−1^ s^−1^). The main mechanisms are radical addition, hydrogen abstraction, and electron transfer.^46^ Another radical species with a similar oxidation potential is the sulphate radical (SO_4_˙^−^),^46^ which transforms OMPs primarily through electron transfer reactions.^47^ Numerous other radical species such as halogen radicals and N-centered radicals also exist, but these play a minor role in the degradation of OMPs, particularly in the presence of other strong oxidants, such as OH-radicals or ozone. Importantly, the assessment of treatability by oxidation processes should consider that OMPs are rarely fully mineralized.^48^ Thus, the transformation products deriving from OMPs and other water constituents formed during oxidation processes should be considered and monitored for their potential to persist and do harm.^14,48^

Oxidants have differing specificities and are capable of oxidizing different reactive moieties within an OMP molecule. To assess their reactivity, OMPs can be broken down into reactive units.^49^ The most important reactive units are aromatic systems, C=C and C=N double bonds, amines and reduced sulfur (Table 1).

**Table 1:** Reactive moieties of micropollutants and their reactivities towards different oxidants^48–54^

Reactive moieties	O_3_	ClO_2_	HOCl	˙OH and SO_4_˙^−^
Aromatic systems	Phenols	Phenols	All	All
	Anilines	Anilines		
Reduced sulfur	Thiols	Thiols	Thiols and Thioether	Thiols
	Thioether			Thioether
	Sulfoxides			Sulfoxides
Double bonds	C=C	Not reactive	Barely	C=C and C=N
Amines	Deprotonated	Tertiary amines deprotonated	Primary > secondary > tertiary amines	All
			Protonated and deprotonated	
C–H	Not reactive	Not reactive	Not reactive	All

The reactive moieties in Table 1 can be present in OMPs with different substituents that can affect the electron density of a reactive moiety. For example, halogenation strongly decreases the reactivity of double bonds towards ozone (*e.g.*, ethene: *k* ∼1.8 × 10^5^ M^−1^ s^−1^, tetrachloroethene: *k* < 10^4^ M^−1^ s^−1^), because halogens withdraw electrons from the double bond.^50^ Other important electron-withdrawing substituents are, C=O, N=C, and COOH/COO–. In contrast, important electron-donating moieties are, *e.g.*, –OH, –NH_2_, methoxy, alkyl, and benzene groups.^55^ When these reactive moieties are incorporated into a molecular structure, the compound is likely to become more amenable to degradation through oxidation processes.

### Current methods and tools for chemical design and their gaps

The effect of individual moieties in molecular structures on the reactivity of a molecule can be estimated by QSARs,^53^ or quantum chemical calculations. Such approaches are useful for screening purposes in the design of new chemicals, after which oxidative treatability and mineralization of promising molecules should always be confirmed experimentally.

In some cases, the potential target moieties of a chemical are so strongly deactivated that they cannot be degraded *via* oxidative processes. An example is the chlorothalonil metabolite, chlorothalonil M12. Here, the reactive moiety is an aromatic 6-membered carbon ring with several electron-withdrawing substituents (2 amides, 2 chlorines and 1 sulfonic group). This makes the benzene ring so electron poor that the reaction rate with ozone drops to a value of < 0.04 M^−1^ s^−1^, and the reaction rate with OH-radical falls below 5 × 10^7^ M^−1^ s^−1^, making these oxidation processes unfeasible.^56^ In some other cases, groups of compounds lack reactive moieties for oxidative attack, as seen with the extremely recalcitrant perfluorinated sulfonates such as perfluorooctane sulfonate (PFOS). These compounds may undergo degradation through electrochemical oxidation initiated by very high potentials.^57^ However, these electrochemical methods are still too energy-intensive to apply in full-scale (waste)water treatment plants without any form of selective preconcentration, and full mineralization is unlikely to occur.

A major fraction of oxidants is consumed by organic matter and other constituents in water, which largely decreases the efficiency of OMP degradation and can lead to the formation of unwanted by-products.^58^ Thus, the effectiveness of oxidation processes is often more dependent on the water matrix than on the molecular structure of target OMPs. Therefore, differences in specificity can outweigh other considerations when selecting oxidants. For instance, SO_4_˙^−^ has a longer half-life and a higher selectivity than ˙OH, leading to a lower interference of dissolved organic matter in SO_4_˙^−^-based oxidation processes.^46^ In fact, SO_4_˙^−^ reacts with humic acids more slowly (6.8 × 10^3^ L per mgC per s) compared to ˙OH (1.4 × 10^4^ L per mgC).^59^ Such extrinsic considerations may need to be taken into account when selecting suitable conditions for measuring treatability *via* oxidation processes.

### Insights for chemical design

Oxidation processes tend to be more resource intensive than other treatment technologies and instead of full mineralization of chemicals, these approaches tend to form transformation products. It is thus crucial to keep the formation of transformation products in mind when designing chemicals meant to be treated *via* oxidation processes based on molecular features detailed in Table 1. Structure-based approaches to predict transformation products, including the abiotic transformation module in Biotransformer^19^ can help identify potential transformation products of concern for a given chemical. To ensure good removal during oxidative processes, reaction rates for target chemicals and their transformation products should exceed 10^8^ M^−1^ s^−1^.

## A hierarchy of chemicals towards a safe future

When considering global inequalities in terms of access to resources, clean water, and (water)treatment technologies, it is evident that the overall production, use, and diversification of chemicals should be curbed.^60,61^ For example, in low- and middle-income economies, conventional wastewater treatment plants are still largely missing (>80% of wastewater remains untreated globally),^62^ let alone advanced water treatment technologies. Thus, safe-by-design chemicals that are non-toxic, readily mineralize in the environment, and are not easily mobilized to groundwater or other susceptible environmental compartments, should be the gold standard. Still, some essential uses for chemicals that are not safe-by-design will remain even if chemical production is curbed and simplified.

**Fig. 4:**
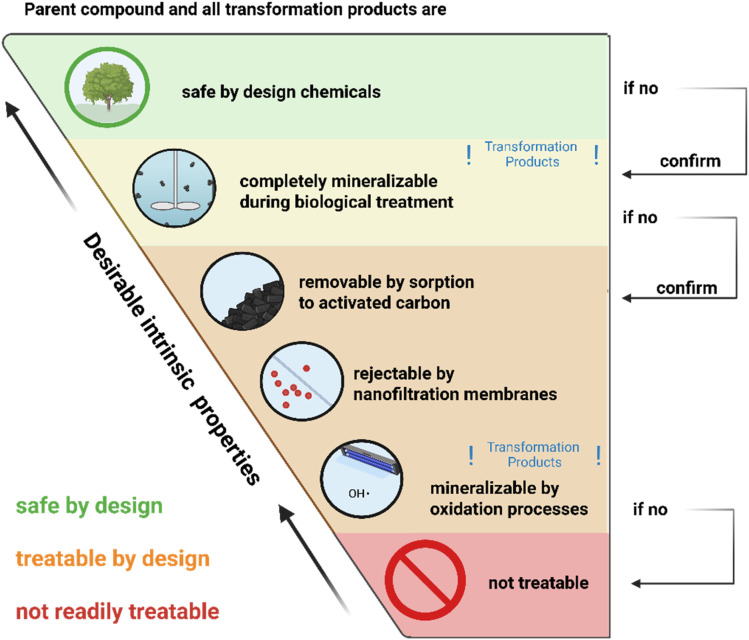
Hierarchy of chemicals, considering their transformation products, towards desirable intrinsic substance properties, from safe-by-design chemicals (top, green), to biologically mineralizable substances (yellow), to substances that are removable *via* advanced treatment technologies (orange), to non-readily treatable substances (red, bottom). The arrows on the right indicate the sequence of considerations that should be made when assessing the profile of a chemical. Formation of transformation products is particularly critical for biological treatments and oxidation processes, labelled with “!Transformation products!” in the figure.

Across the treatment technologies described above, the treatability of a given chemical and its transformation products differ not only among different compounds, but also in terms of the feasibility for different water matrices, monetary costs, and environmental impacts (*e.g.*, resource use, emissions, *etc.*). Consequently, based on these factors and more, we propose a hierarchy of treatability by specific technologies towards desirable intrinsic chemical properties, as illustrated in Fig. 4, aiming to minimize human health and environmental impacts.

For chemicals, where the desired use conflicts with safe-by-design (*e.g.* for pharmaceuticals that need to be stable in a patient's body), the hierarchy of treatabilities shown in Fig. 4 could be followed. Biological treatment under differing redox conditions towards complete mineralization would be the preferred treatability type with the lowest energy and resource use profile (yellow field in Fig. 4), followed by the more laborious and costly advanced separation and oxidation techniques which may be ranked differently based on water composition and target chemical (orange field in Fig. 4). Therein, AC production and regeneration are energy- and CO_2_ intensive, but typically have a lower environmental footprint than NF, which relies on continuous energy supply for operating pumps and produces concentrate streams that need additional treatment. Oxidative processes also rely on continuous energy supply for oxidant production, and their environmental footprint is often higher compared to both AC and NF.^63,64^ Hazardous chemicals with unknown treatability, or hazardous chemicals that fall into the “non-treatable” field (red in Fig. 4), should be avoided. In cases where such non-treatable chemicals are currently essential, they should be prioritized for substitution and phase-out. The same holds for chemicals forming “non-treatable” transformation products. Thereby, the formation of transformation products is of particular importance during biological treatment and oxidative processes, whereas this is less of an issue for separation-based technologies.

To implement the hierarchy of treatability-by-design, molecular features discussed in the previous sections can inform the selection from the wide range of already existing chemicals, as well as the design of future chemicals. When designing such compounds, it is important to ensure no persistent and non-readily treatable transformation products are formed. If the necessary properties of a designed chemical cannot accommodate biodegradability, complete removal *via* separation and/or chemical oxidation processes should be ensured based on molecular features related to adsorption affinity, removal *via* NF, and/or complete mineralization in oxidation processes. Treatment trains that combine approaches from the orange and yellow fields are commonly implemented in some high-income countries and can constitute a strong barrier that makes most chemicals treatable to some extent. In some cases, combining *e.g.* separation and destruction-based technologies can lower the overall cost of treatment.^65^ Still, when assessing the treatability in the context of treatment trains, the additional monetary and environmental costs always need to be considered in a holistic manner, considering both implementation and operation of a given treatment. In practice, the number and type of treatment train constituents should generally be minimized to decrease operational complexity, financial, and environmental impacts. To curb monetary and environmental costs, prioritizing on-site treatment of OMP hotspot streams, *e.g.*, industrial or hospital waste streams,^66^ could make a substantial positive impact on human and ecosystem health, even when no generalized water treatment is available. Introducing chemicals that are readily treatable, ideally by biological processes, would be crucial to make such approaches practicable.

Further expanding and integrating knowledge on the molecular properties driving treatability into an integrated framework will allow a comprehensive assessment of treatability as discussed above. Such a framework could inform and guide future regulation and efforts,^8^ ultimately resulting in improved development, assessment and management of chemicals towards safe-by-design (preferred) and treatable-by-design chemicals. Our analysis here provides a starting point for developing such a framework by the wider scientific, engineering and regulatory communities.

## Author contributions

GS: conceptualization, visualization, writing – original draft, writing – review & editing, SJS: writing – original draft oxidation section, AG: writing – original draft separation section, TVW: writing – original draft biodegradation section, MA: writing – review & editing, BMA: writing – review & editing, JED: writing – original draft separation section, HVL: writing – original draft oxidation section, MN: writing – review & editing, NBS: writing – original draft biodegradation section, DZ: writing – review & editing, ZW: writing – original draft, writing – review & editing.

## Conflicts of interest

There are no conflicts to declare.

## Data Availability

No new data were created or analysed in this study. Data sharing is not applicable to this article
